# Chimeric antigen receptors enable superior control of HIV replication by rapidly killing infected cells

**DOI:** 10.1371/journal.ppat.1011853

**Published:** 2023-12-15

**Authors:** Yuqi Zhou, Julie Jadlowsky, Caitlin Baiduc, Alex W. Klattenhoff, Zhilin Chen, Alan D. Bennett, Nicholas J. Pumphrey, Bent K. Jakobsen, James L. Riley

**Affiliations:** 1 Department of Microbiology and Center for Cellular Immunotherapies, Perelman School of Medicine, University of Pennsylvania, Philadelphia, Pennsylvania, United States of America; 2 Adaptimmune Ltd, Abingdon, United Kingdom; 3 Immunocore Ltd., Abingdon, United Kingdom; Vaccine Research Center, UNITED STATES

## Abstract

Engineered T cells hold great promise to become part of an effective HIV cure strategy, but it is currently unclear how best to redirect T cells to target HIV. To gain insight, we generated engineered T cells using lentiviral vectors encoding one of three distinct HIV-specific T cell receptors (TCRs) or a previously optimized HIV-targeting chimeric antigen receptor (CAR) and compared their functional capabilities. All engineered T cells had robust, antigen-specific polyfunctional cytokine profiles when mixed with artificial antigen-presenting cells. However, only the CAR T cells could potently control HIV replication. TCR affinity enhancement did not augment HIV control but did allow TCR T cells to recognize common HIV escape variants. Interestingly, either altering Nef activity or adding additional target epitopes into the HIV genome bolstered TCR T cell anti-HIV activity, but CAR T cells remained superior in their ability to control HIV replication. To better understand why CAR T cells control HIV replication better than TCR T cells, we performed a time course to determine when HIV-specific T cells were first able to activate Caspase 3 in HIV-infected targets. We demonstrated that CAR T cells recognized and killed HIV-infected targets more rapidly than TCR T cells, which correlates with their ability to control HIV replication. These studies suggest that the speed of target recognition and killing is a key determinant of whether engineered T cell therapies will be effective against infectious diseases.

## Author summary

Infusion of HIV-specific engineered T cells is a promising approach to eliminate HIV infected cells that reverse latency and begin to replicate HIV after ART withdrawal. However, it is unclear whether chimeric antigen receptors (CAR) or TCRs are the best approach to generate HIV-specific T cells for adoptive T cell therapy. We generated CD8 T cells expressing one of three HIV-specific TCRs (TCR-T) or a previously optimized HIV-specific CAR and interrogated which population controlled HIV replication more effectively. HIV-specific CAR T cells were far superior to any of the TCR-T cells. Increasing TCR affinity or interfering with the ability of Nef to downregulate MHC class I had modest effects on the ability TCR-T cells to control HIV. Inserting additional cognate epitopes within the HIV genome did improve the ability of TCR-T cells to control HIV but HIV CAR T cells were still superior. The ability to control HIV replication best correlated with the ability of engineered T cell to rapidly induce cell death in HIV-infected T cells, suggesting that the speed of recognition and initiation of T cell mediated killing is a key determinant on whether engineered T cells can control viral infections.

## Introduction

The latent HIV reservoir remains a significant barrier to an HIV cure [1]. “Shock and Kill” is an approach intended to reduce the reservoir by using an agent to induce HIV gene expression within the latently infected cells, thus making these cells visible to agents that can specifically target and eliminate them [2]. Much effort has focused on agents to jolt HIV out of latency [3], whereas less attention has focused on an agent that could rapidly recognize and eliminate these infected cells [4]. In people living with HIV (PLWH) who have controlled HIV replication long-term by antiretroviral therapy (ART), the natural HIV-specific immune system is not well positioned to serve as a killing mechanism and this may explain the lack of significant reservoir reduction after “Shock and Kill” clinical trials [5]. T cell exhaustion, immune escape mutations, and effective ART render low numbers of functional HIV-specific cells [6]. As such, an infusion of highly active anti-HIV agents has been proposed to bolster the HIV-specific immune response prior to the initiation of a “Shock and Kill” therapeutic strategy [7].

Because of their durability, specificity, and effector functions, genetically modified T cells are an attractive “Kill” agent [8]. However, it is unclear how best to engineer HIV-specific T cells to maximize their effectiveness. Part of this uncertainty is due to our incomplete understanding as to why some people have more effective natural T cell responses than others. Nearly all individuals generate robust T cell responses to HIV, but the magnitude of this response does not correlate with control of HIV replication [9]. HIV has evolved effective means to avoid natural immune responses including the ability of Nef to downregulate HLA class I [10]. Nonetheless, there is a small population of PLWH who are able to control HIV replication in the absence of ART (elite controllers) [11]. Elite controllers, as a population, are highly enriched for a select number of HLA class I alleles including HLA-B57, suggesting that CD8 T cells play an important role in controlling HIV replication in these rare individuals. However, why these T cells can durably suppress HIV replication while the vast majority of HIV-specific T cells cannot remains to be fully elucidated [12]. Several studies of elite controllers demonstrate that polyfunctionality [13], resistance to Treg suppression [14], generation of escape mutations that result in increased sensitivity to restriction factors [15], preferentially targeting Gag epitopes [16], and high avidity [17,18] are all correlated with elite control of HIV replication. However, these studies often compare cohorts of patients whose HLA types, viral swarm, time since infection, and overall health status can vary considerably, making side-by-side comparisons difficult. Here, we address the question of what properties best associate with control of HIV replication by engineering the same T cells with different re-targeting genes, bypassing some of the confounding issues seen when studying T cell responses from different individuals.

Currently, it is unclear what is the best way to redirect T cells to recognize HIV infected cells. One approach genetically modifies T cells by introducing TCRs that recognize HIV-specific peptides presented within specific HLA molecules, which imparts the ability to target the whole HIV genome, to sense minute levels of peptide/HLA on the cell surface, and to leverage the natural T cell signaling apparatus [19]. However, a TCR can recognize many peptides presented by the HLA [20], and tragic outcomes have occurred when an introduced TCR recognizes an unexpected self-peptide [21]. Moreover, HLA restriction requires the development of multiple TCRs so that most PLWH could benefit from this therapy, and even further engineering to reduce expression of endogenous TCR genes to prevent even more off-target effects [22]. HIV-specific CAR T cells, on the other hand, use targeting domains that are very specific for the HIV envelope, thus minimizing concerns for undesired T-cell-mediated killing. Furthermore, CAR T cells would be less affected by immune evasion strategies [23]. Here, our studies demonstrate that the time by which redirected T cells recognize and initiate killing is a quality that best predicts the control of HIV replication and suggest CAR T cells will be more effective than TCR T cells in HIV cure studies.

## Results

### TCR and CAR T cells targeting HIV-specific antigens have similar functional profiles

**Fig 1:**
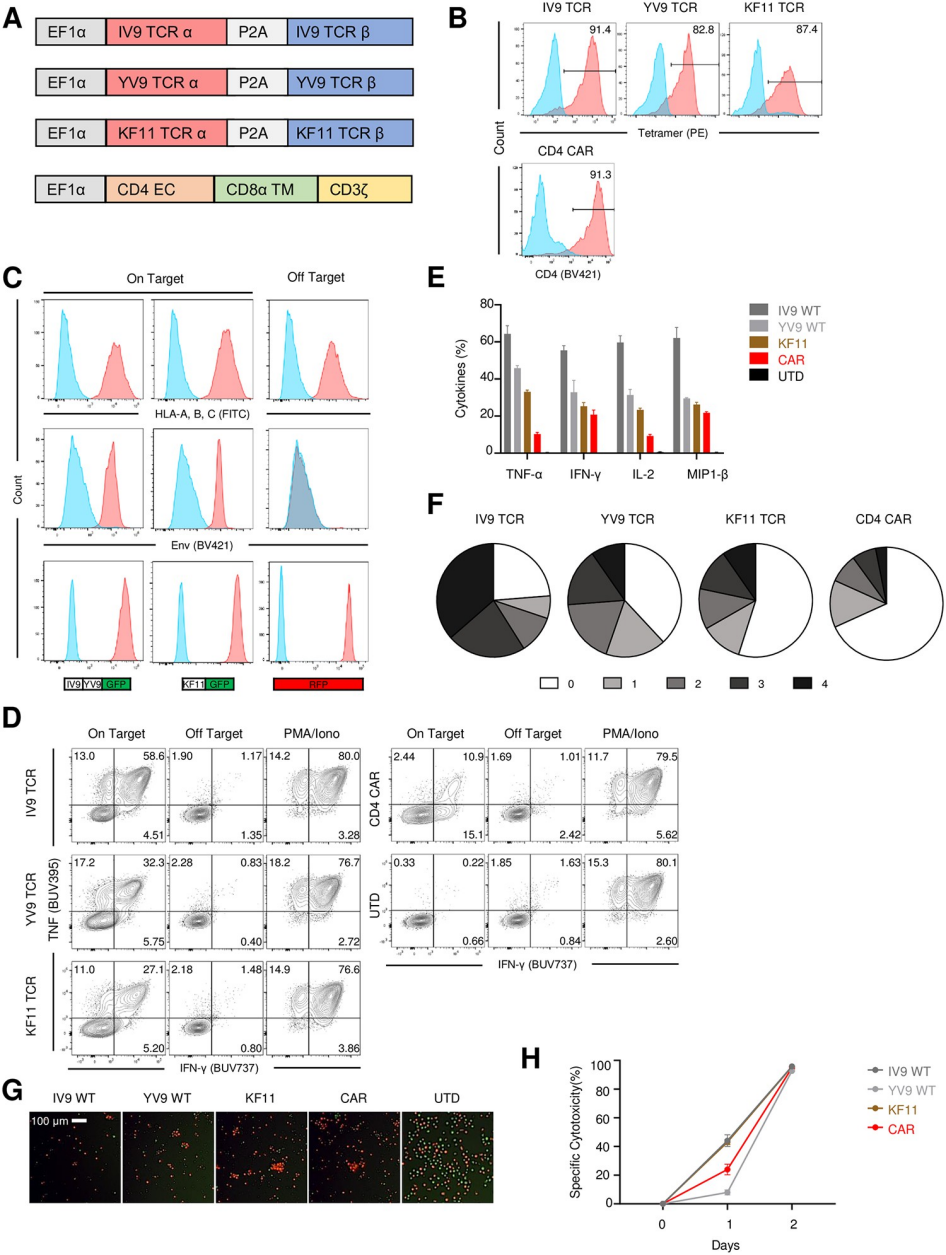
TCR and CAR T cells targeting HIV-specific antigens have similar functional profiles. **A.** Schematic drawing of constructs used to generate HIV specific engineered T cells. All constructs were driven by the EF-1α promoter and a P2A sequence was used to allow co-expression of the indicated TCRα and TCRβ chain genes. **B.** Purified human CD8 T cells were activated, transduced with a lentiviral vector encoding A2-IV9 TCR, A2 YV9 TCR or a CD4 zeta CAR, cultured for 6 days, and stained using a tetramer (IV9 and YV9 TCR), anti-Vβ 13 antibody (KF11), or anti-CD4 antibody. **C-D.** After 8 days of culture, TCR or CAR engineered T cells were incubated with either on target and off-target aAPCs (**C**) for 4 hours and intracellular cytokine production of IL-2, TNFα, IFN-γ and MIP1-β were measured by flow cytometry (**D**). PMA and ionomycin was used as a positive control for cytokine production. **E.** Summary data from 3 independent experiments from **D. F.** Pie chart shows the percentage of T cells producing 1 or more cytokines. **G-H.** Equal numbers of on target and off target aAPCs from **C**. were mixed with the indicated engineered T cell population or untransduced T cells (UTD) at a 1:1 ratio for 24 hours and the resulting aAPC populations were visualized by flow cytometry (**G**) or image cytometry **(H**). I. Specific cytotoxicity was calculated by (1—Average on target aAPC cell number with engineered T cells/ Average on target aAPC cell number with UTD). Error bars represent SD.

To study the ability of an array of engineered HIV-specific T cells to control HIV replication, we compared our previously optimized CD4-based CAR [24] whose only signaling domain was CD3 zeta to allow for fairer comparison with the following array of TCR T cells targeting the following peptide-HLA (pHLA) complexes: A2-IV9 (Pol_476-484_) is an HLA-A2 immunodominant response whose presence is not correlated with HIV control [25]; A2-YV9 is an HLA-A2 subdominant, rare response that targets the highly conserved catalytic domain of Pol_181-189_ [26]; and B57-KF11 (Gag_162-172_) is a HLA-B57 restricted TCR whose presence is correlated with lower viral loads and is present in many elite controllers [27]. Sequences encoding these TCRs were placed in lentiviral vectors (Fig 1A), and these vectors were used to transduce anti-CD3/28 activated primary human CD8 T cells (Fig 1B). To assess the functional properties of these engineered T cells, we generated K562-based artificial antigen-presenting cells (aAPCs) [28] that expressed either HLA-A2 with a minigene that expressed ~50 amino acids surrounding the A2-IV9 and ~50 amino acids surrounding A2-YV9 epitope linked with GFP, or HLA-B57 and a minigene surrounding ~50 amino acids of the B57-KF11 epitope linked with GFP. Both of these on-target aAPCs expressed truncated Env [24]. An aAPC that expresses HLA-A2, HLA-B57 and mCherry was also constructed to serve as antigen off-target control (Fig 1C). Each engineered T cell population was mixed with an on target aAPC, off target aAPC or treated with PMA and ionomycin, and their ability to produce cytokines or kill targets in a specific manner was measured (Figs 1D–1H and S1A–S1C). We observed all engineered T cell populations produced robust cytokine responses and killed antigen-specific targets but not closely related off-target cells. Of note, the CAR T cells produced less cytokines than the TCR T cells in response to on target aAPC co-culture, and this is due the lack of a costimulatory domain [24,29]. Moreover, in this aAPC model, the immunodominant A2-IV9 and B57-KF11 T cells were able to kill targets faster than A2-YV9 and the CD4-based CAR T cells but ultimately all target cells were eliminated. In summary, all the engineered T cells were able to induce robust responses in an antigen-specific manner.

### CD4-based CAR T cells control HIV replication better than TCR T cells

**Fig 2:**
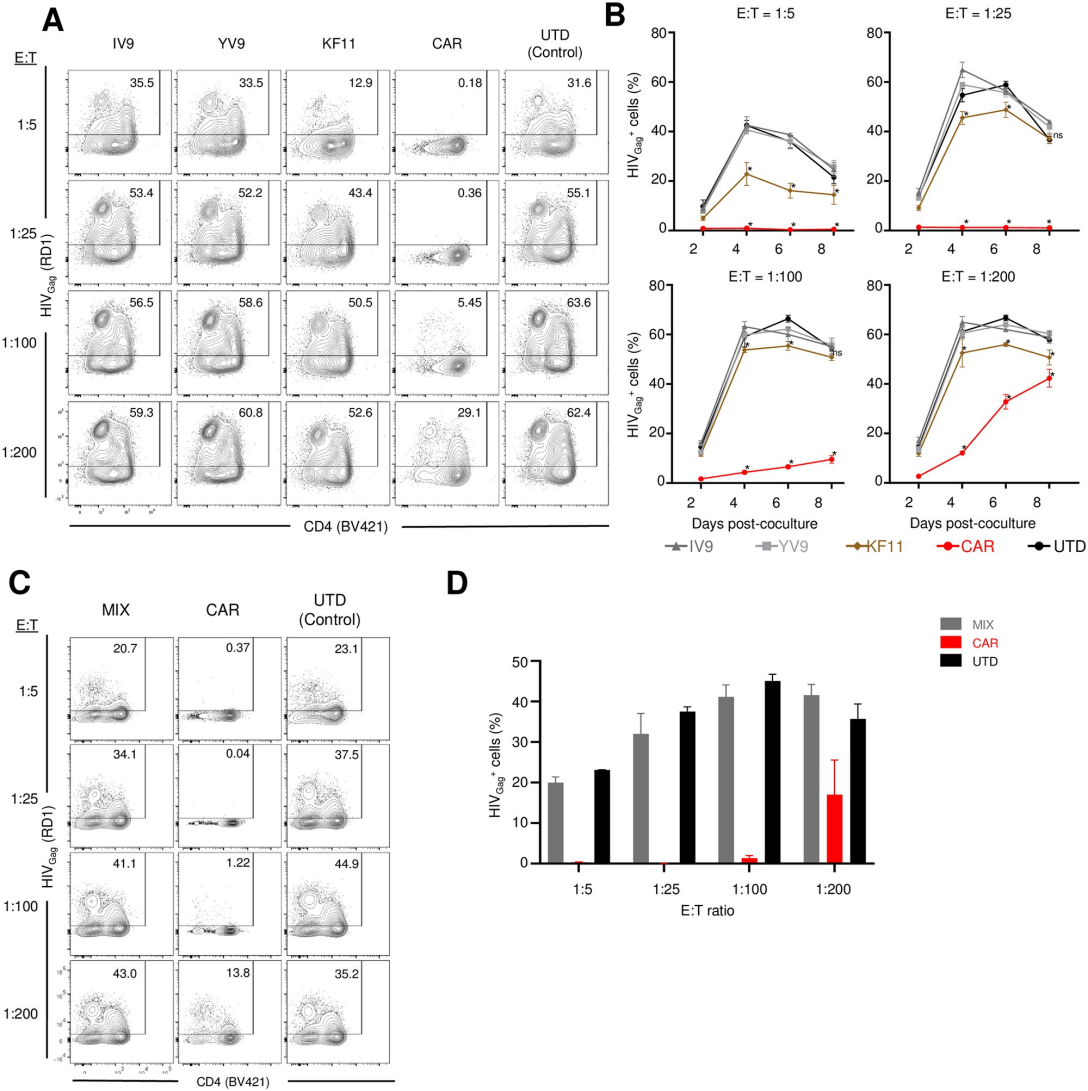
CD4 CAR T cells control HIV replication better than TCR T cells. **A-B.** Primary CD4 T cells from an HLA-A2^+^ HLA-B57^+^ donors were activated with CD3/28 beads and cultured for 6 days before being infected with HIV_NL4-3_. The following day, these infected CD4 T cells were then cocultured with the indicated TCR or CAR engineered T cells at the indicated ratio. Intracellular staining for Gag was performed every other day. Flow cytometry plot shows representative data 6 days post co-culture and **B.** summarizes data from one experiment performed in triplicate. **C-D.** IV9, YV9, and KF11 TCR T cells were mixed equally and compared with CD4 CAR T cells for their ability to suppress HIV replication. **D.** Bar graph shows summarized data of **C.** 6 days post coculture. Results are representative of three independent experiments in which each condition was performed in triplicate. Error bars represent SD. Significance was determined through 1-way ANOVA test (p values: ns >0.05, *<0.05).

We next asked which of these engineered T cells was best able to control HIV replication. To do this, we mixed each engineered CD8 T cell at a range of E:T ratios with HIV infected CD4 T cells and monitored the ability of the CD8 T cells to control the spread of HIV within the culture. Compared to non-transduced CD8 T cells, A2-IV9- and A2-YV9- specific T cells had no ability to control HIV replication. B57-KF11 specific T cells were able to reduce HIV replication at high E:T ratios but not at low E:T ratios. In contrast, CD4-based CAR T cells were able to effectively reduce HIV replication at a 1:100 E:T ratio (Figs 2A, 2B, and S2A). To better detect the activity of the TCR-transduced T cells, we reduced the inoculum of HIV used to infect the CD4 T cells by 10-fold. We observed that B57-KF11 specific T cells were able to show better control of HIV replication at high E:T ratios but neither A2-IV9 or A2-YV9 specific T cells demonstrated any anti-HIV activity despite the lower inoculum (S2B and S2C Fig). We hypothesized that a mixture of TCR-transduced T cells would be able to control HIV replication better than a single TCR-transduced population. However, this is not what we observed, and this mixture was unable to control HIV replication (Figs 2C, 2D, and S2D), highlighting the inability of either A2-YV9 or A2-IV9 specific T cells to control HIV replication. This data shows that despite similar functional profiles against aAPCs, the ability of these engineered T cells to control HIV replication varied widely.

### Affinity enhancement of Pol-specific TCRs retain specificity, confer co-receptor independence, and recognize common escape mutants but fail to augment control of HIV-1 replication

**Fig 3:**
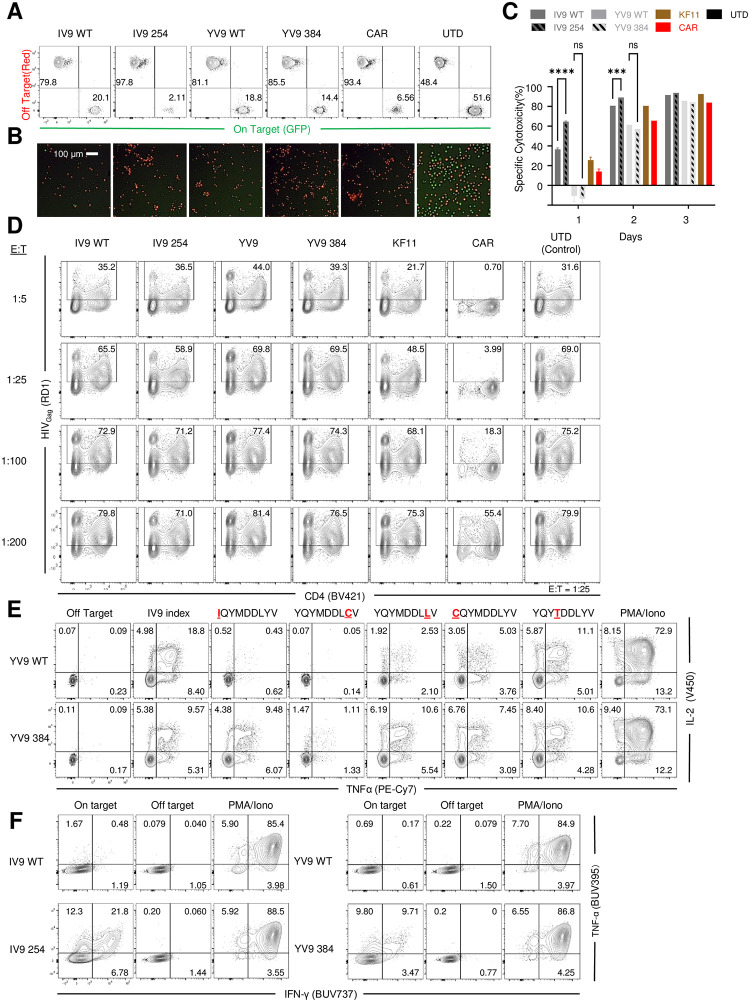
Affinity enhancement of Pol-specific TCRs retain specificity, confer co-receptor independence, and recognize common escape mutants but fail to augment control of HIV-1 replication. **A-C.** Primary human CD8 T cells were activated and either left untransduced (UTD) or transduced with lentiviral vectors encoding A2-IV9 TCR, an affinity enhanced A2-IV9 TCR (254), A2-YV9 TCR, an affinity enhanced A2-YV9 TCR (384), and a CD4 CAR. After 6 days of culture, 500,000 T cells were co-cultured with 250,000 on target (GFP) and 250,000 off-target (mCherry) aAPCs and measured for the ratio of GFP vs mCherry cell using flow cytometry (**A**) or image cytometry (**B**). Summary data of **A** from three independent experiments. Specific cytotoxicity was calculated and error bars indicate standard deviation (SD). Significance was determined through 1-way ANOVA test (p values: ns >0.05, *** <0.001, **** < 0.0001). **D.** Activated CD4 T cells were infected with HIV_NL4-3_ for 24 hours and then cocultured with the indicated engineered or untransduced T cells at 25:1 target to effector ratio to measure their ability to suppress HIV spread within the culture. Flow cytometry shows CD4 expression and intracellular Gag of Live /CD8- cells at Day 6 post coculture. **E.** Primary human CD4 T cells expressing WT and affinity enhanced A2-IV9 or A2 YV9 specific TCRs were cocultured with on or off target K562 cells for 4 hours and intracellular staining for IFNγ and TNFα was performed. PMA and ionomycin was used as a positive control for cytokine production. **F.** Primary human CD8 T cells expressing WT and affinity enhanced A2 YV9 specific TCRs were cocultured with K562.A2.B57 aAPCs that were loaded with index (WT) and the indicated mutated YV9 peptide for 4 hours and intracellular IL-2 and TNFα was measured by flow cytometry.

Previously, we observed that increasing TCR affinity for pMHC complexes augmented the ability of TCR T cells to recognize immune escape mutants and control HIV replication [30]. To determine whether augmenting the affinity of A2-IV9 and A2-YV9 TCR increased their ability to control HIV replication, we used phage display (A2-YV9) or rationale design mutagenesis (A2-IV9) approaches to generate a library of affinity enhanced TCRs whose affinity was augmented up to 20-fold (S3A Fig). We ascertained both the specificity and function of these affinity enhanced TCRs and chose one TCR pair from each family that conferred the most polyfunctionality and target specificity (A2-IV9 254 and A2-YV9 384, S1 Table and S3B and S3C Fig). Using our aAPC-based model of killing, we observed that affinity enhancement of A2-IV9 and to a lesser extent A2-YV9 increased the TCR T cells’ ability to specifically kill targets (Fig 3A–3C). However, when we asked if the affinity enhanced TCRs could control HIV replication better than T cells expressing the wildtype TCRs, we saw no significant change in control of HIV replication (Figs 3D and S3D). Since we observed no improvement in the ability of the affinity enhanced TCRs to control HIV replication, we wanted to determine whether our affinity enhanced TCRs had properties associated with other TCRs that went through affinity enhancement. For instance, affinity enhanced TCRs often can recognize closely related epitopes that the wildtype TCR cannot recognize, which can have beneficial effects such as seeing HIV escape mutants [30] or tragic outcomes such as recognizing self-tissue [21]. A2-YV9 targets the catalytic domain of Pol and since many antiretroviral drug escape mutations are in this region, we asked whether A2-YV9 384 recognized some of the ART escape mutants. Indeed, we observed that this affinity enhanced TCR was able to recognize or better recognize IQYMMDDLYV, YQYMDDLLV, YQYMDDLCV and CQYMDDLYV escape mutants that confer resistance to Nevirapine [31] and Efavirenz [32], respectively (Fig 3E). Additionally, TCR affinity enhancement often allows T cells to recognize and respond to peptide/HLA in a co-receptor independent manner [33,34] which can improve clinical outcomes [35]. We observed that both affinity enhanced A2-IV9 and A2-YV9 TCRs were able to confer HLA class I specificity to CD4 T cells (Fig 3F). In summary, although TCR affinity enhancement was able to improve effector function, the ability to recognize HIV escape mutants, and confer HLA class I recognition to CD4 T cells, affinity enhancement was unable to improve the ability of A2-IV9 and A2-YV9 TCR T cells to control the spread of HIV replication.

### Lack of HLA downregulation helps TCR T cells recognize HIV infected T cells but does not enable control of HIV replication

**Fig 4:**
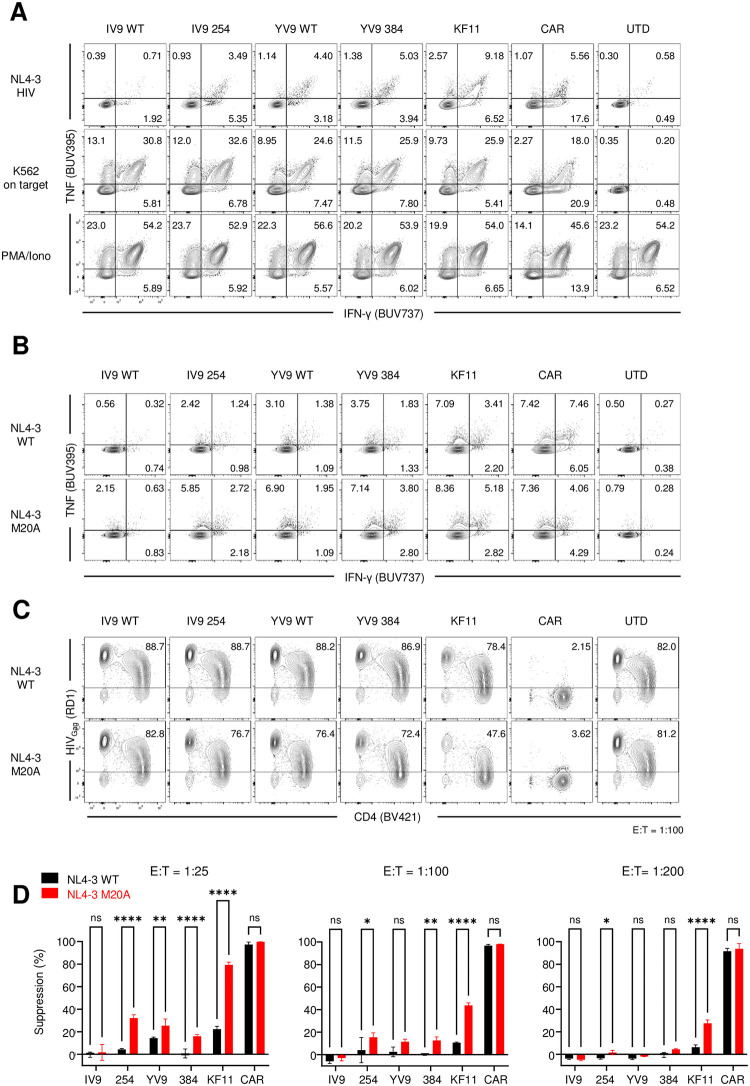
Lack of HLA downregulation helps TCR T cells recognize HIV infected T cells but does not enable control of HIV replication. **A.** Primary CD4 T cells were activated and infected with HIV_NL4-3_ on the following day. 4 days after infection, when roughly 50% of the CD4 T cells were Gag positive, the indicated engineered or untransduced CD8 T cells (UTD) were cocultured with infected CD4 T cells at a 1:1 ratio for 6 hours and stained intracellularly for IFNγ and TNFα. On target aAPCs and PMA + ionomycin were used as positive controls. **B.** Engineered T cells were cocultured with activated CD4 T cells infected with HIV _NL4-3_ or HIV_NL4-3_ Nef M20A and stained intracellularly for cytokines. **C.** CD4 T cells were infected with HIV _NL4-3_ or HIV_NL4-3_ Nef M20A for 24 hours and then cocultured with the indicated engineered or untransduced T cells at 100:1 target to effector ratio to measure their ability to suppress HIV spread within the culture. Flow cytometry shows CD4 expression and intracellular Gag of Live /CD8- cells at Day 6 post coculture. **D.** Summarized triplicate data from C using the indicated E:T ratio. Results are representative of three independent experiments with different donors performed in triplicate. Error bars indicate standard deviation (SD). Significance was determined through 1-way ANOVA test (p values: ns >0.05, * <0.05, **< 0.01, *** <0.001, **** <0.0001).

The strength of antigen signal can dictate which functional properties T cells exhibit [36,37], so we were curious to determine whether TCR T cells were able to recognize HIV infected T cells but simply unable to remove the infected cell before it could spread the virus to other CD4 T cells. To determine the ability of TCR T cells expressing A2-IV9 and A2-YV9 wildtype and affinity enhanced TCRs to recognize HIV-infected cells, we first infected primary human CD4 T cells with HIV for 3 days, making sure a large proportion of the cells were infected (S4 Fig), then co-cultured engineered HIV-specific T cells for 4 hours with these HIV infected cells or with the aAPCs used in Fig 1C, and measured the intracellular cytokine response. As before, all engineered T cells gave robust responses when mixed with aAPCs expressing the relevant antigen with IV9 254>IV9 WT> YV9 384>YV9 WT>KF11>CAR whereas control CD8 T cells produced background cytokine responses. Surprisingly, all but IV9 WT TCR T cells responded to HIV infected cultures (Figs 4A and S5A) but the strength of response differed from what was seen with aAPCs with CAR>KF11>YV9 384>YV9 WT>IV9 254>IV9 WT. Nef can downregulate HLA class I expression [38] and thereby dampen HIV-specific CD8 T cell responses and HLA-B57 seemed more resistant to this downregulation than other HLA alleles such as HLA-A2 [39]. Previously, it was demonstrated that a single mutation (M20A) in Nef interfered with HLA class I downregulation [40,41]. To delineate the degree by which HLA class I downregulation interfered with TCR T ability to recognize and eliminated HIV infected cells, we compared engineered T cells to recognize and control both wildtype and Nef M20A HIV. We observed that most of the TCR T cells produce more cytokines to Nef M20A infected CD4 T cells than wildtype infected cells. Curiously, A2-IV9 WT TCR T cells could not recognize either virus well (Figs 4B and S5B). Expectedly, CD4-based CAR T cell recognition was not altered by this mutation in Nef since CAR recognition is Nef independent. This improved recognition of Nef M20A infected CD4 T cells did result in slightly better control of HIV replication even by IV9 WT TCR T cells but in no case did the potency match HIV CAR T cells (Figs 4C, 4D, and S8).

### Insertion of an extra A2-IV9 and A2-YV9 epitope improves the ability of TCR T cells to control HIV replication but CD4-based CAR T cells are still superior

**Fig 5:**
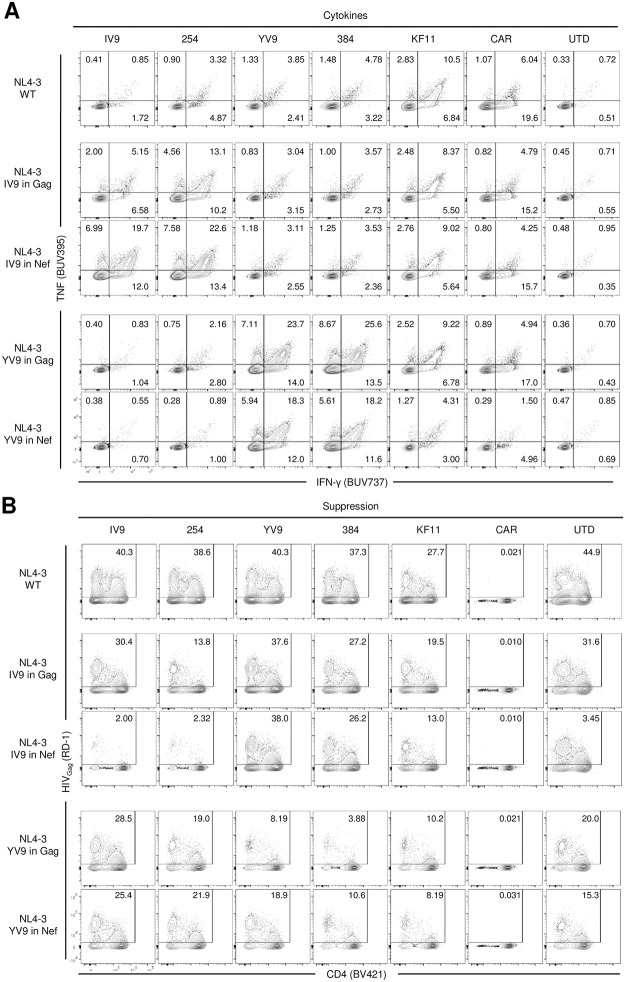
Insertion of an extra A2-IV9 and A2-YV9 epitope improves the ability of TCR T cells to control HIV replication but HIV CAR T cells are still superior. **A.** Activated human CD4 T cells were infected with HIV_NL4-3_ or with the indicated epitope inserted HIV_NL4-3._ The indicated TCR and CAR T cells with mixed with the infected CD4 T cells at a 1:1 ratio for 6 hours and intracellular TNFα and IFNγ was measured by flow cytometry. **B.** Primary human CD8 T cells (UTD) or those transduced with wildtype and affinity enhanced A2-IV9 or A2 YV9 specific TCRs or CD4 CAR T cells were cocultured with HIV infected CD4 T cells at the 1:100 ratio. Intracellular Gag staining was performed 6 days after co-coculture. Results are representative of three independent experiments performed in triplicate collected at multiple time points.

Since the level of recognition and cytokine expression of the engineered HIV-specific T cells mirrored their ability to control HIV replication, we hypothesized that insertion of an additional epitope within the HIV genome would augment the amount of peptide/HLA reaching the cell surface and bolster both the cytokine response and control HIV replication by the TCR T cells. To do this, we added either the A2-IV9 and A2-YV9 epitope in Gag [42] and to the C- terminus of Nef (S7A Fig). These viruses were used to infect CD4 T cells, and we first measured the ability of the engineered cells to recognize HIV infected cells in culture. As in Fig 4, B57-KF11 specific and CAR T cells gave strong responses to HIV infected cultures. The addition of IV9 in Gag and more so in Nef lead to robust recognition of the A2-IV9 wildtype TCR transduced T cells and even more so with A2-IV9 254 TCR transduced T cells, whereas no differences in A2-YV9 specific T cells was noted, indicating that the improved recognition is due to the inserted A2-IV9 epitope (Figs 5A and S6). Likewise, the A2-YV9 TCR transduced T cells had substantially augmented recognition of HIV carrying an extra YV9 epitope; however, neither the location of the extra YV9 epitope nor the affinity of A2-YV9 TCR altered this enhanced recognition. To determine whether this augmented T cell recognition translated to improved control of HIV replication, we asked if these strains of HIV containing an extra epitope of the target antigens were controlled better by the respective TCR-transduced T cell population. Indeed, where robust cytokine responses were observed, control of HIV replication was also noted. However, CD4-based CAR T cells still controlled HIV replication better than TCR-transduced T cells, suggesting that factors other than robust recognition of HIV infected T cells are enabling CD4-based CAR T cells to exert improved control of HIV replication (Figs 5B and S9).

### CAR T cells control HIV through rapid induction of active Caspase 3 in HIV infected cells

**Fig 6:**
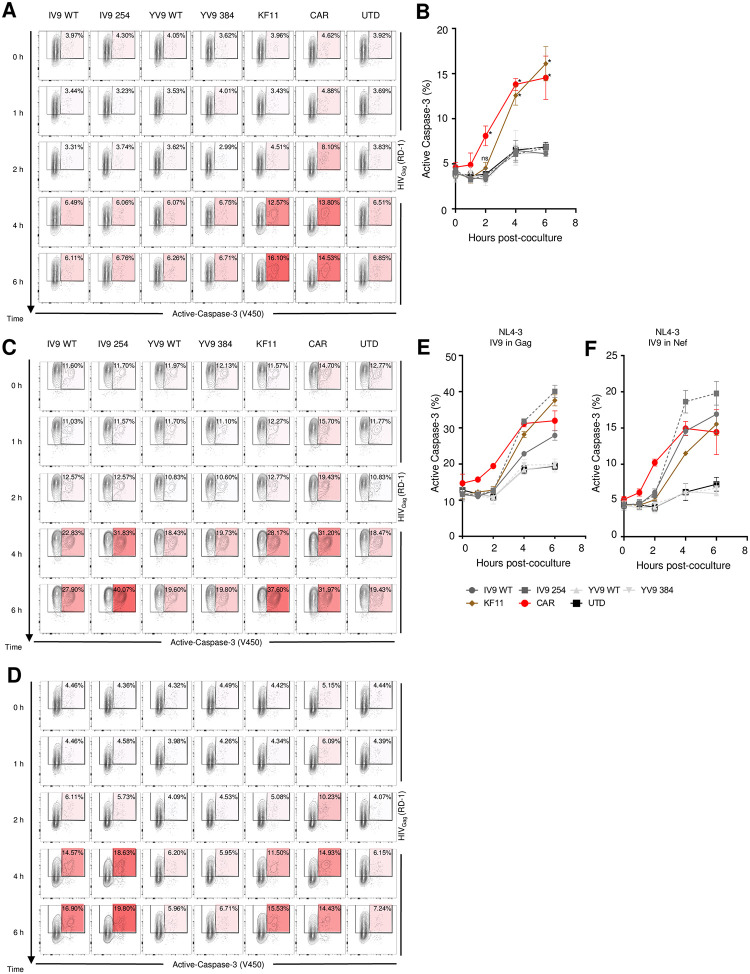
CAR T cells control HIV through rapid induction of active Caspase 3 in HIV infected cells. **A-B.** Human CD4 T cells were activated and infected with HIV_NL4-3_ for 4 days. Infected CD4 T cells were cocultured with the indicated engineered T cells at 1:1 ratio. both intracellular active Caspase 3 and Gag were measured at the indicated time points after co-culture. Numbers on flow plot show percentage of active Caspase 3 expressing cells in Gag^+^ CD8- cells. The ratios are also displayed in heatmap overlapped on flow plot ranging from light (low) to dark red (high). **B.** Line graph shows summarized data from three independent experiments over the course of time. **C-F.** Activated human CD4 T cells were infected with IV9 in Gag (**C.**) or IV9 in Nef (**D.**) HIV for 4 days. Infected CD4 T cells were cocultured with untransduced or the indicated engineered T cells at 1:1 ratio. Both intracellular active Caspase 3 and Gag were measured at the indicated time points after co-culture. **E-F**. Line graphs show summarized data from three independent experiments over the course of time.

We hypothesized that the speed by which CD4-based CAR T cells initiate death pathways in HIV infected targets could explain their superior ability to control HIV replication since the longer an infected cells is able to produce daughter virions, the more HIV will spread. Induction of active Caspase 3 is an irreversible step in CTL mediated cell death and can serve as a sensitive marker for the induction of cell death [43]. We infected CD4 T cells with HIV, mixed in engineered CD8 T cells, and measured the induction of active Caspase 3 over time. Since these were infected CD4 T cells, there were 3–4% of the T cells expressing active Caspase 3 and this percentage increased to ~6% over the next 6 hours when non-transduced, A2-IV9, and A2-YV9 were mixed into the culture, suggesting that the addition of A2-IV9 and A2-YV9 TCR T cells did not augment the number of HIV infected T cells dying (Fig 6A and 6B). In contrast, cultures containing CD4-based CAR had significantly more active Caspase 3 expressing T cells after 2 hours and those cultures containing B57-KF11 TCR T cells had more CD4 T cells expressing active Caspase 3 after 4 hours of co-culture. The data show a correlation between induction of Caspase 3 in infected CD4 T cells and control of HIV replication and suggests that CD4-based CAR can kill infected targets faster than TCR-transduced T cells. To further explore this, we performed the same assay using HIV that contained M20A mutation in Nef. Here, B57-KF11 TCR transduced T cells killed the HIV-infected cultures over background after 2 hours of cultures and we were able to observe some augmented activity of the affinity enhanced A2-IV9 and A2-YV9 after 6 hours of co-culture. As with the HIV suppression assays in Fig 4, CD4-based CAR T cells were able to induce above background levels of active Caspase 3 after 1 hour of co-culture (S10 Fig). Lastly, we performed a similar assay using the HIV strains that had extra A2-IV9 epitopes since whether the extra epitope was placed in Gag or Nef altered the ability of A2-IV9 TCR-transduced T cells to control its replication (Fig 5). Here, we observed that more rapid killing by the A2-IV9 and A2-IV9 254 TCR transduced T cells that mirrored what we observed in the HIV suppression assay (Figs 6C–6F and S11). Collectively, these findings underscore that the capacity of TCR and CAR T cells to inhibit HIV spread is dependent on their ability to rapidly induce cytotoxicity in HIV infected targets.

## Discussion

Engineered T cell therapies for cancer have highlighted both the potency and durability of this approach, prompting many to consider engineered T cells as part of an HIV cure strategy [23,44]. Immune escape, T cell exhaustion, and the requirement for durable persistence are shared challenges facing both HIV and cancer CAR T cells [45]. However, there are several unique challenges when targeting engineered T cells at HIV including minute levels of antigen after infusion of T cells into PLWH controlling HIV using ART, the susceptibility of T cells to HIV infection, loss of CD4 T cell helper functions, and the rate and mechanism by which HIV can spread [46]. A key question is how to best redirect T cells to recognize HIV to address the unique challenges HIV presents. Both *ex-vivo* expanded HIV-specific CAR T [47–55] and TCR T cells (both engineered and natural) [18,30,56–64] have been tested for their ability to target HIV and one study compared CTL clones with an early version of the CD4 CAR [65]; however, the studies described here are the first to compare CARs and TCRs in primary T cells side-by-side with the goal of providing insight into which one works better and why.

CARs have many features that make them attractive to use in HIV cure strategies including the lack of HLA restriction, insensitivity to Nef, and the ability to engineer multiple specificities into a single construct [66]. However, a study that engineered both a CAR and TCR T cell to recognize the same peptide/HLA found that TCR T cells were at least a 100-fold more sensitive [67]. Selection of CD19 low tumors in patient treated with CD19-specific CARs indicate that this lack of sensitivity of CAR T cells could help enable immune escape [68]. For HIV, this lack of sensitivity toward target may be more of an issue than in cancer since considerable spread of HIV to nearby cells could occur prior to the infected cells being recognized by the CAR. HIV envelope is the only HIV protein expressed on the cell surface and thus the only target for CAR T cells. As a target, it is not ideal. Env has a remarkable track record of avoiding immune targeting as its structure can accommodate a large array of sequence variation [69]. Additionally, rapid recycling limits the amount of Env on the cell surface [70], making sensitivity an even more important issue. Lastly, HIV Env is Rev dependent so it is not rapidly translated by the newly infected cell and thus unlikely to be an early beacon of HIV infection [71]. Given these limitations, it was surprising that CD4-based CAR T cells were so much more effective than HIV-specific TCR T cells. Moreover, we underestimated the potency by which CAR T cells control HIV replication by not adding a costimulatory domain so that we could more directly compare the ability of CAR and TCRs to redirect T cells to HIV without confounding the issue by having the CAR and TCR T cells linked to distinct signaling pathways. CD4-based CARs with costimulatory domains certainly have more potency than those lacking costimulatory signals [24,72,73].

To understand why the CAR T cells were more effective than the TCR T cells in controlling HIV replication, we made a number of modifications to the experimental system to see what factors would allow the TCR T cells to work as well as CAR T cells. Augmenting TCR affinity often results in improved T cell sensitivity and function [74], but we did not see any improvements in the ability of affinity enhanced A2-IV9 and A2-YV9 TCR T cells to control HIV replication despite clear improvements in T cell sensitivity and function. This suggested that efficient HIV spread was occurring prior to the point when these TCR T cells were able to recognize and kill HIV infected targets. To further investigate this, we engineered HIV to express an additional A2-IV9 and A2-YV9 epitope in both Rev dependent (Gag) and independent proteins (Nef) [75]. Using these viruses, we were able to observe control of HIV replication by the TCR T cells. Both wildtype and affinity enhanced A2-IV9 TCR T cells controlled viral replication when the extra A2-IV9 epitope placed in Nef; however, only the affinity enhanced A2-IV9 specific TCR T cells robustly controlled HIV replication when the extra epitope was placed in Gag, suggesting that higher affinity TCRs are better able to control antigens produced in a Rev dependent manner in some cases, but not all, as we saw no differences in the ability A2-YV9 specific T cells based on epitope location or TCR affinity. Nonetheless, we did observe significant control of these epitope-translocated viruses, indicating that these infected T cells were presenting sufficient levels of the relevant peptide/HLA on their surface in time for the TCR T to eliminate them before significant viral spread could occur. While interfering with Nef’s ability to downregulate HLA class I expression did have an effect, it was modest. This suggests that the rate of antigen processing to present sufficient peptide/HLA on the cell surface to trigger TCR T cell killing is the most important factor in deciding TCR T control of HIV infection rather than the absolute level of HLA class I on the cell surface.

So then, why do CAR T cells control HIV replication better than TCR T cells? Previous work has shown that CAR T cells form a different immunological synapse that enables faster killing than natural TCR-based antigen recognition [76]. Moreover, there are two major routes of HIV spread: cell-free and cell-associated. Cell-free infection would require *de novo* production of HIV Env and thus probably might be less well controlled by CAR T cells. However, cell to cell transmission involves HIV Env transfer from the productively infected T cell to the newly infected T cell [77]. Thus, our data would support a model that a CAR T cell could kill a recently infected T cell right after HIV Env is transferred from the donor to the recipient, and this would certainly be an effective way of preventing HIV spread. It is currently unclear how much HIV spread *in vivo* is driven by cell associated versus cell free mechanisms *in vivo*, but most would agree that cell to cell transmission is responsible for a significant fraction of HIV spread [78]. However, for an effective HIV cure strategy one may need a strategy to block cell free spread (i.e. broadly neutralizing Abs) and CAR T cells to block cell to cell transmission. There are some caveats to our study that are worth noting. For one, we did not block expression of the endogenous TCR genes. This has been shown to both improve the specificity and sensitivity of TCR-engineered T cells by limiting mispairing of TCR chains [79–81]. However, blocking the expression of endogenous TCR also improves the activity of CAR T cells [82] so it is unclear on whether disabling the expression of endogenous would have improved the ability of TCR-engineered T cells to control HIV replication relative to CD4-based CAR. Additionally, comparing TCR and CAR single molecule affinity for a target via surface plasmon resonance ignores the complicated biology of TCR recognition and signaling [83], and thus it is unclear from our studies whether CD4-based CAR T cells or TCR-engineered T cells bind HIV infected targets with more affinity/avidity. In summary, these studies support using the speed by which an agent kills an HIV-infected target as part of the criteria by which agents are selected for HIV Cure studies.

## Materials and methods

### Plasmid construction and generation of lentiviral vectors and HIV viral stocks

Lentiviral transfer vectors encoding A2-IV9 and B57-KF11 specific TCRs and CD4 (CD8 TM) zeta chain CAR have been previously described [24,84]. Sequences for TCRα and β chain genes encoding for A2-YV9 were generously provided by Dr. June Kan-Mitchell [85] and placed into pTRPE [86] to generate pTRPE A2-YV9 TCRα T2A A2-YV9 TCRβ. pTRPE B57 was constructed by obtaining a gene block containing the coding sequence of HLA-B57 (GenBank AAA36231.1) from IDT with appropriate restriction sites and placing this gene block into pTRPE backbone. pELNS HLA-A2 [28] and the pTRPE truncated YU.2 envelope expression construct [24] have been previously described. Lentiviral supernatants were produced as previously described and stored at -80°C until needed [72]. pNL4-3 plasmid-encoded HIV strains containing extra A2-IV9 and A2-YV9 epitopes were synthesized by Reniguard Life Sciences (Exton, PA). S6 Fig shows where the sequences were inserted. Replication competent HIV was generated by harvesting supernatants from 293 T cells transfected with these constructs as previously described [87].

### Protein expression and purification

Procedures used for preparation of proteins used in this study have been described [88]. Peptides were obtained from Peptide Protein Research Ltd. (Fareham, UK). Codon-optimized genes for all the proteins used in this study, including soluble forms of both TCR α and β chains, soluble β2m (residues 21–119), and a soluble HLA–A*02:01 heavy chain (residues 25–276), were cloned into the pGMT7 expression vector (Promega, Southampton, UK). Soluble HLA-A*02:01 heavy chain was expressed with a C–terminal in vitro biotinylation tag and refolded in the presence of both soluble β2m and peptide. After enzymic biotinylation with BirA (Avidity, Colorado, CA), this refolded complex was purified as soluble HLA-biotinylated peptide HLA (peptide/HLA) monomers by ion exchange and gel filtration to PBS. Refolding of soluble TCR α/β heterodimer was assisted by an artificial disulfide bond introduced by genetic engineering [88].

### Generation of affinity-enhanced TCR mutants and biochemical characterization

A2-YV9 affinity-enhanced TCR mutants were engineered by using parental TCR α and β chains as templates for mutagenesis of their complementarity-determining regions. High-affinity mutants were selected by phage display panning with peptide/HLA-coated magnetic beads as previously described [30]. For A2-IV9, a rationale design approach was taken by which mutations that increased the affinity of a similar TCR were grafted into the A2-IV9 TCR. Mutations from specific binders were cloned as separate TCR α and β chains and refolded for affinity analysis. Equilibrium dissociation constants (KD) between TCRs and relevant biotinylated peptide/HLA monomers were determined as described using streptavidin–coupled CM5 sensor chips and a Biacore3000 instrument (GE Healthcare, Little Chalfont, UK) [88].

### Primary human T cell culture, T cell transduction, and HIV infection

De-identified, HLA typed and purified human CD4 and CD8 T cells were obtained from the University of Pennsylvania’s CFAR and Human Immunology Core (HIC RRID: SCR_022380). Both subsets of T cells were cultured in CTS OpTmizer T-cell expansion SFM(Gibco) with 1% Penicillin-Streptomycin, 2mM GlutaMax and 25mM HEPES buffer, and 100U/ml IL-2 at 1x10^6^ cells per ml. Cell culture flasks were incubated at 37° C, 5% CO_2_ and 95% humidity. T cells were stimulated with anti-CD3/CD28 Dynabeads (Life Technology) at 3:1 ratio. 18 hours after stimulation, appropriate amount of CAR or TCR lentiviruses were added into the culture of CD8 T cells. 48 hours after stimulation, the culture volume was quadrupled with culture medium. At 4 days after activation, the anti-CD3/CD28 beads were removed using a magnet. Throughout the culture after bead removal, medium was supplied every 2 days to maintain a cell concentration of 0.5x10^6^ cells per ml.

### Flow cytometry

Cells were washed with PBS containing 2mM EDTA and 2% FBS, resuspended in the same PBS solution and stained with Biolegend antibodies: CD3(OKT3), CD45(2D1), CD4(OKT4), CD8(RPA-T8 and SK-I), HLA-A2(BB7.2), HLA-ABC(W6/32), TCRVb13.1(H131);

BD Bioscience: CD8(SK-1), CD4(RPA-T4); MBL International: iTAg Tetramer/PE—HLA-A*02:01 HIV Pol (ILKEPVHGV), QuickSwitch HLA-A*02:01 Tetramer Kit-PE or tetramers obtained from NIH Tetramer Core Facility. Cell viability was identified through Fixable Viability Dye eFlour 780 (eBioscience). Specific cell populations were identified using Cell Trace Violet or Cell Trace Far-Red (Thermo Fisher Scientific). Detection of intracellular protein was accomplished through BD Fixation/Permeabilization Solution Kit. The following antibodies from BD were used IL2 (MQ1-17H12), MIP1β (d21-1351), IFNγ (4S.B3), TNFα (MAB11), Active Caspase 3 (C92 605). The HIV core Antigen antibody (KC57) was obtained from Beckman Coulter. To measure the production of intracellular cytokine, 1×10^5^ TCR or CAR T cells were cocultured with 2×10^5^ aAPCs. One hour after coculture, 1x Brefeldin A and Monensin Solution (Biolegend) was added. After 6 hours of total incubation at 37°C, cells were harvested and stained intracellularly for TNF-α, IFN-γ, MIP-1β, IL-2 and analyzed by flow cytometry.

### aAPC generation and antigen-specific killing assay

K562 cells were transduced with lentiviral vectors and single cell sorted. Clones expressing uniform levels of each marker were chosen. The specific cytotoxicity of TCR and CAR T cells was measured by the elimination of on-target aAPCs relative to off-target aAPCs. In a 96 well plate 5 × 10^4^ TCR or CAR T cells were co-cultured with 5 × 10^4^ of the relevant aAPCs. The total number of K562 cells were identified with Celigo Image Cytometer (Nexcelom) at 0h, 24h, and 48h. Specific killing was calculated based on percent of cells killed with the presence of TCR or CAR T cells as previously described [89].

### HIV Suppression assay

The ability of engineered T cells to prevent HIV spread has been previously described [73]. Briefly, HLA-A2 and HLA-B57 CD4 and CD8 T cells were activated with anti-CD3/CD28 coated beads. The CD8 T cells were transduced with either an HIV-specific CAR or TCR the following day and then cultured for 7 days. The CD4 T cells were infected with HIV_NL4-3_ and the following day non-transduced and engineered CD8 T cells were mixed at various ratio. HIV p24 core antigen was then measured by flow cytometry every other day to measure the rate of HIV spread throughout the culture.

### HIV infected T cell killing assay

HLA-A2 and HLA-B57 CD4 T cells were activated with anti-CD3/28 beads and infected with HIV_NL4-3_ or epitope added variants. Once ~20–30% of the CD4 T cells were HIV infected, engineered T cells were mixed in and the percentage of CD4 T cells expressing active Caspase 3 was determined by flow cytometry.

### Statistical analysis

Graphpad Prism version 9.5 was used for all statistical analysis.

## Supporting information

S1 Fig **A-C.** Data from two other experiments (Donor#2 and Donor#3) replicating the data shown in Fig 4E–4H.
(TIF)

S2 Fig **A.** Summarized HIV suppression data from two separate donors (Donor#2 and Donor#3). See Fig 2B for details. **B.C.** Primary CD4 T cells from an HLA-A2^+^ HLA-B57^+^ donor were activated with CD3/28 beads and cultured for 6 days before being infected with 1/10 dose of HIV_NL4-3_. The following day, these infected CD4 T cells were cocultured with the indicated TCR or CAR engineered T cells at the indicated ratio. Intracellular staining for Gag was performed every other day. Representative flow cytometry plot after 6 days of co-culture (**B.**) and summarized data for the entire experiment (**C**). **D.** Replicate experiments from Fig 2C.
(TIF)

S3 Fig **A.** Disassociation constant (KD) of soluble wildtype and affinity enhanced A2-IV9- and A2 YV9-specific TCRs was made by Biacore. **B.** Primary human CD8 T cells were activated and left alone (Untransduced) or transduced with WT or affinity enhanced IV9 (mutant 254) and YV9 (mutant 384) which were the affinity enhanced TCRs that showed the most potency and maintained specificity. 6 days after the transduction IV9 or YV9 tetramer were used stain the untransduced (blue) or the indicated TCR-transduced (red) T cells and staining was measured by flow cytometry. **C.** Polyfunctional pie chart shows WT and affinity enhanced IV9 and YV9 TCR producing 1 or more cytokines (IL-2, IFN-γ, TNF-α, MIP-1β). **D.** Summarized HIV suppression data for Fig 3D (Donor#1) and two other representation experiments (Donor #2 and #3).
(TIF)

S4 Fig Primary CD4 T cells were activated and infected with HIV_NL4-3_ WT or HIV_NL4-3_ Nef M20A on the following day. 4 days after infection CD4 surface staining and intracellular staining of Gag was performed and analyzed by flow cytometry.
(TIF)

S5 Fig **A.** Summary intracellular cytokine staining data for Fig 4A is shown for 3 independent experiments. **B.** Summary intracellular cytokine staining data for Fig 4B is shown for 3 independent experiments. Note these experiments were performed at the same time which is why the data for HIV_NL4-3_ WT is duplicated.
(TIF)

S6 Fig Summary intracellular cytokine staining data for Fig 5A is shown for 3 independent experiments. (TIF)

S7 Fig **A.** Epitope inserted HIV_NL4-3_ was made by addition of the indicated epitope sequence within pNL4-3 plasmid. Amino acid sequence of the edited spot is shown. **B and C.** Primary CD4 T cells were activated and infected with HIV_NL4-3_ WT or indicated mutant viruses on the following day. 4 days after infection, intracellular Gag and surface CD4 expression (**B.**) or intracellular Gag and HLA-A2 surface staining **(C.)** was performed. Blue color represents cells infected with HIV_NL4-3_ WT and red depicts cells infected with the indicated HIV_NL4-3_ mutant.
(TIF)

S8 Fig Summarized suppression assay for two additional separate donors described in Fig 4D. (TIF)

S9 Fig Summarized data for three donors from the experiment described in Fig 5B. (TIF)

S10 Fig **A**. Intracellular active Caspase 3 was measured after the coculture of engineered T cells and CD4 T cells infected with HIV_NL4-3_ Nef M20A for the indicated time (hours). **B.** Summarized data from **A.**
(TIF)

S11 Fig Summarized intracellular active Caspase 3 staining data for two additional separate donors described in Fig 6E and 6F. (TIF)

S1 Table Amino acid sequence of WT and affinity enhanced IV9 and YV9 TCRs. (TIF)
