# Supplementary figures and images for: Chimeric antigen receptors enable superior control of HIV replication by rapidly killing infected cells
Source: PLoS Pathog. 2023 Dec 15;19(12):e1011853. doi: 10.1371/journal.ppat.1011853 (PMC10773964; doi:10.1371/journal.ppat.1011853)

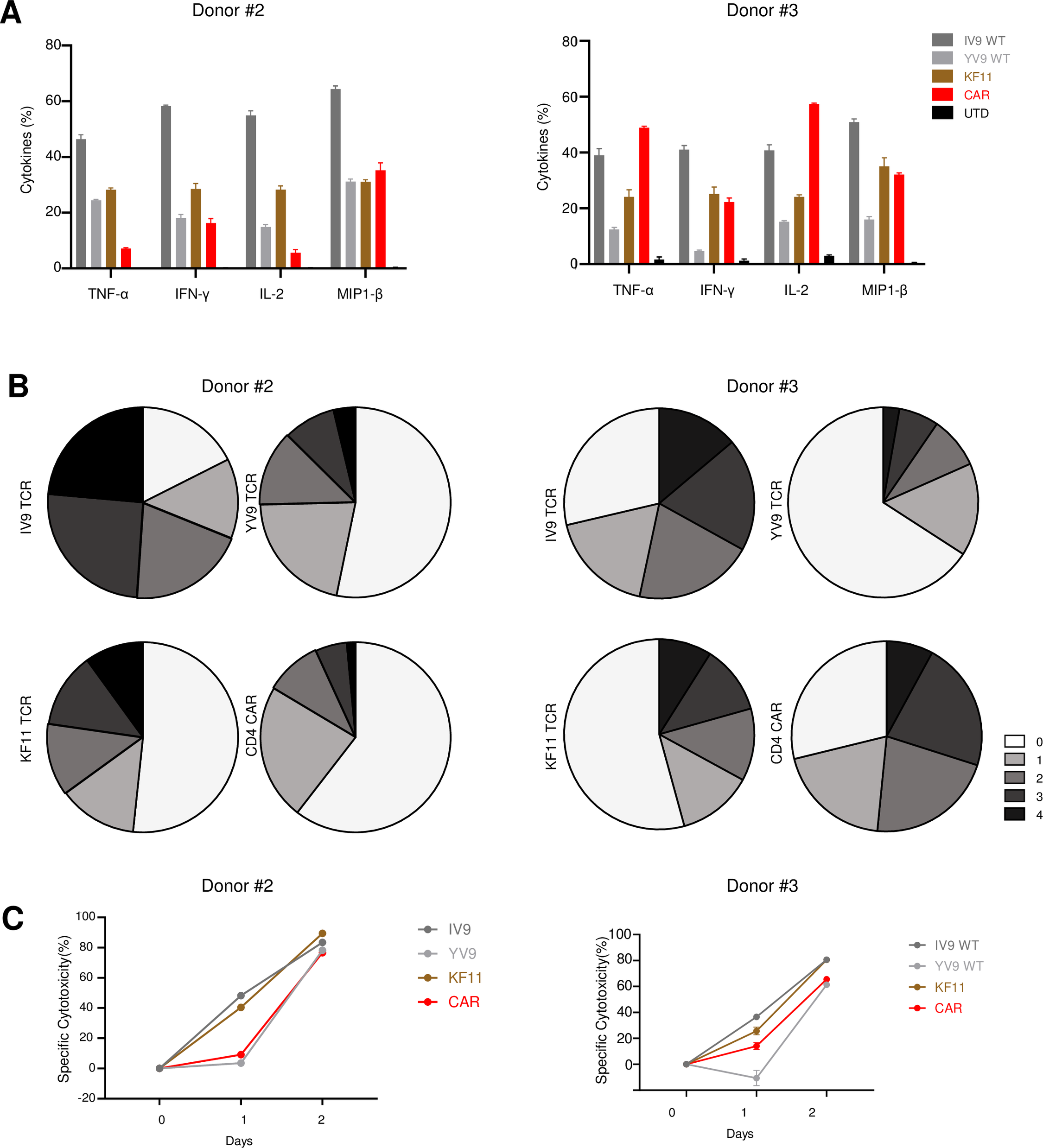

Supplement: S1 Fig — A-C. Data from two other experiments (Donor#2 and Donor#3) replicating the data shown in Fig 4E–4H. (TIF) [file ppat.1011853.s001.tif]

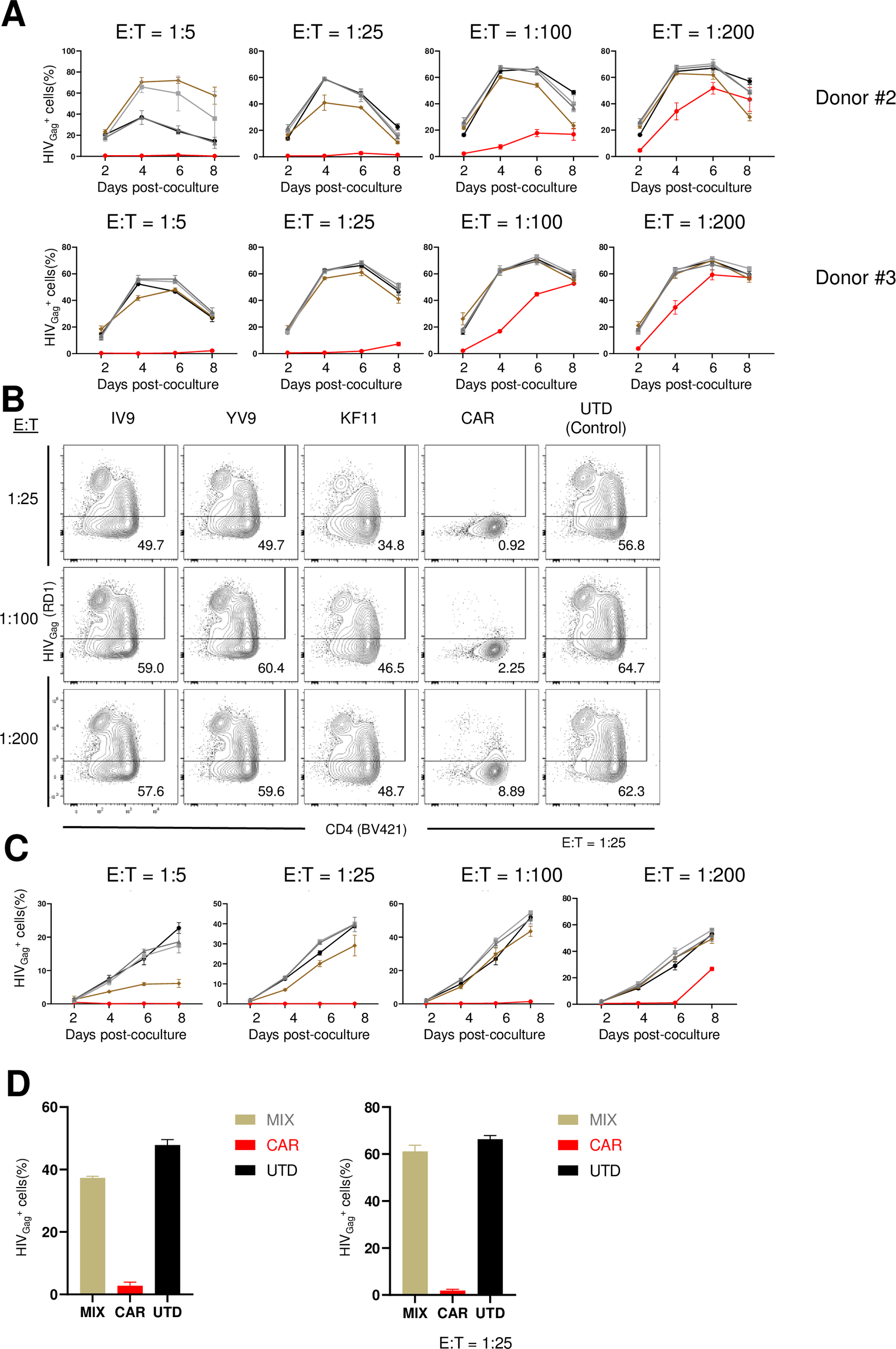

Supplement: S2 Fig — A. Summarized HIV suppression data from two separate donors (Donor#2 and Donor#3). See Fig 2B for details. B.C. Primary CD4 T cells from an HLA-A2+ HLA-B57+ donor were activated with CD3/28 beads and cultured for 6 days before being infected with 1/10 dose of HIVNL4-3. The following day, these infected CD4 T cells were cocultured with the indicated TCR or CAR engineered T cells at the indicated ratio. Intracellular staining for Gag was performed every other day. Representative flow cytometry plot after 6 days of co-culture (B.) and summarized data for the entire experiment (C). D. Replicate experiments from Fig 2C. (TIF) [file ppat.1011853.s002.tif]

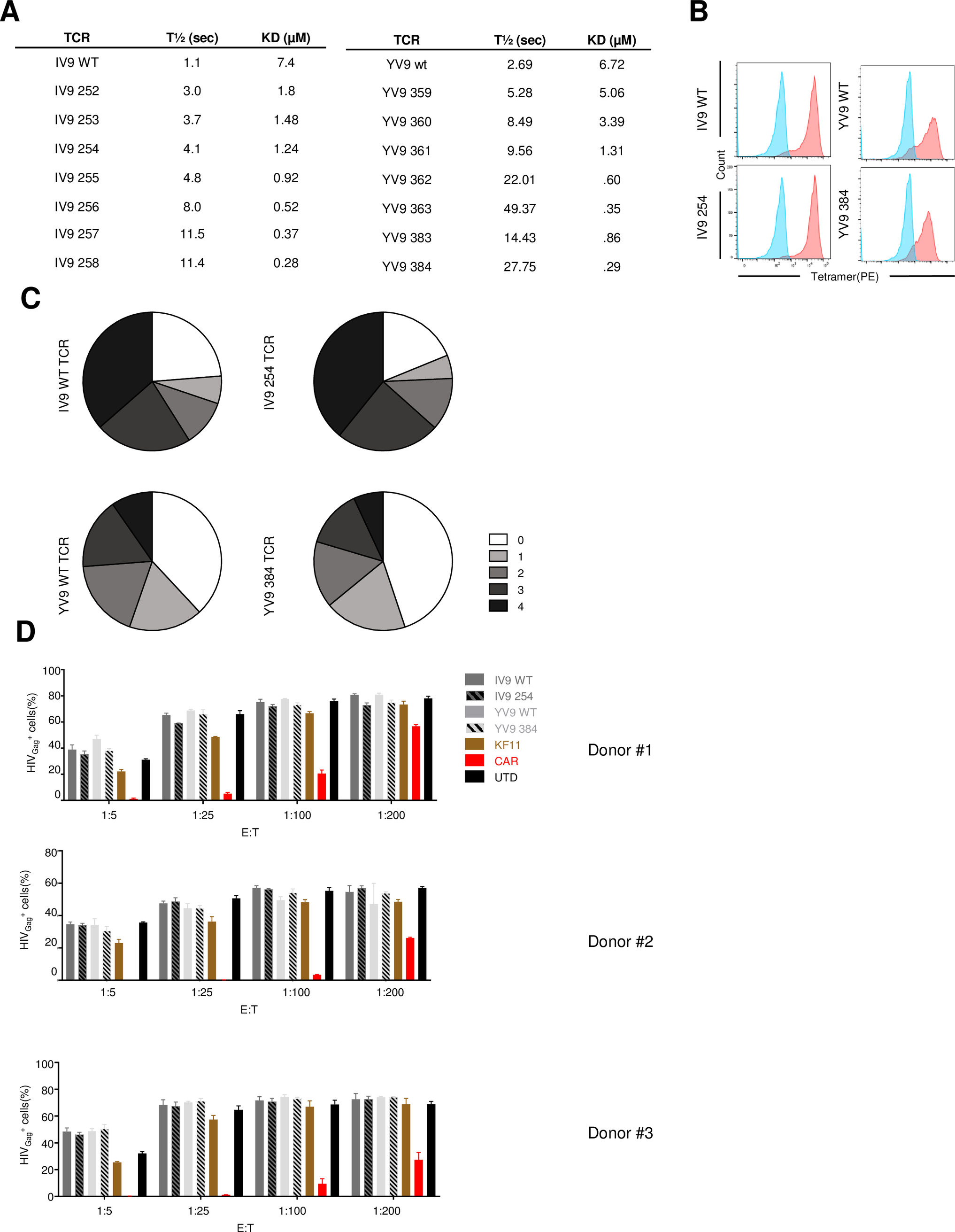

Supplement: S3 Fig — A. Disassociation constant (KD) of soluble wildtype and affinity enhanced A2-IV9- and A2 YV9-specific TCRs was made by Biacore. B. Primary human CD8 T cells were activated and left alone (Untransduced) or transduced with WT or affinity enhanced IV9 (mutant 254) and YV9 (mutant 384) which were the affinity enhanced TCRs that showed the most potency and maintained specificity. 6 days after the transduction IV9 or YV9 tetramer were used stain the untransduced (blue) or the indicated TCR-transduced (red) T cells and staining was measured by flow cytometry. C. Polyfunctional pie chart shows WT and affinity enhanced IV9 and YV9 TCR producing 1 or more cytokines (IL-2, IFN-γ, TNF-α, MIP-1β). D. Summarized HIV suppression data for Fig 3D (Donor#1) and two other representation experiments (Donor #2 and #3). (TIF) [file ppat.1011853.s003.tif]

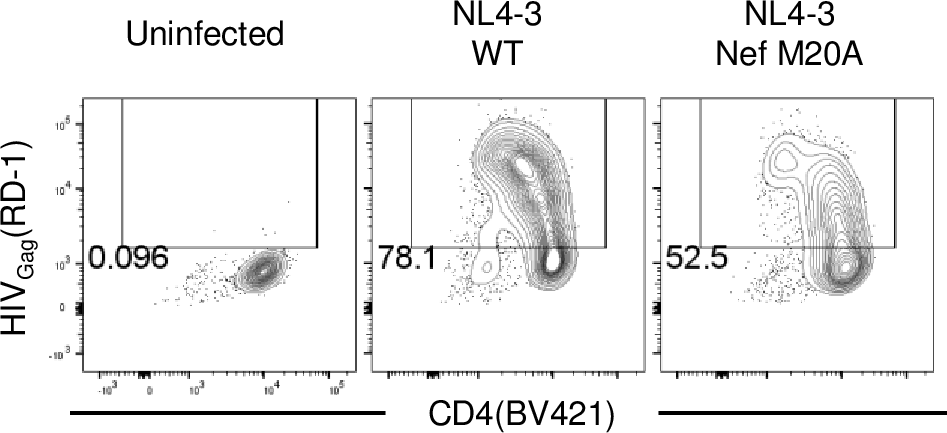

Supplement: S4 Fig — 4 days after infection CD4 surface staining and intracellular staining of Gag was performed and analyzed by flow cytometry. (TIF) [file ppat.1011853.s004.tif]

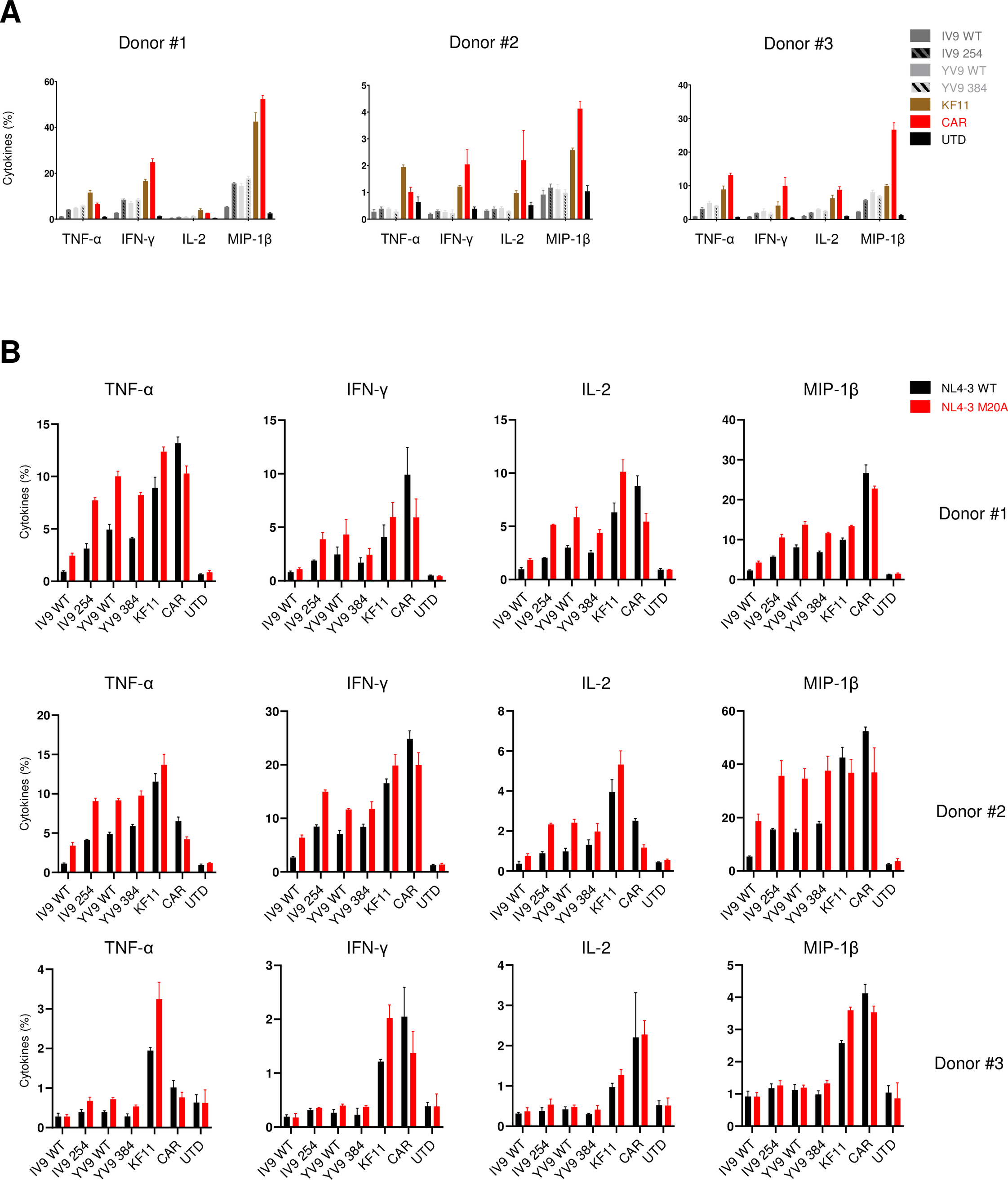

Supplement: S5 Fig — A. Summary intracellular cytokine staining data for Fig 4A is shown for 3 independent experiments. B. Summary intracellular cytokine staining data for Fig 4B is shown for 3 independent experiments. Note these experiments were performed at the same time which is why the data for HIVNL4-3 WT is duplicated. (TIF) [file ppat.1011853.s005.tif]

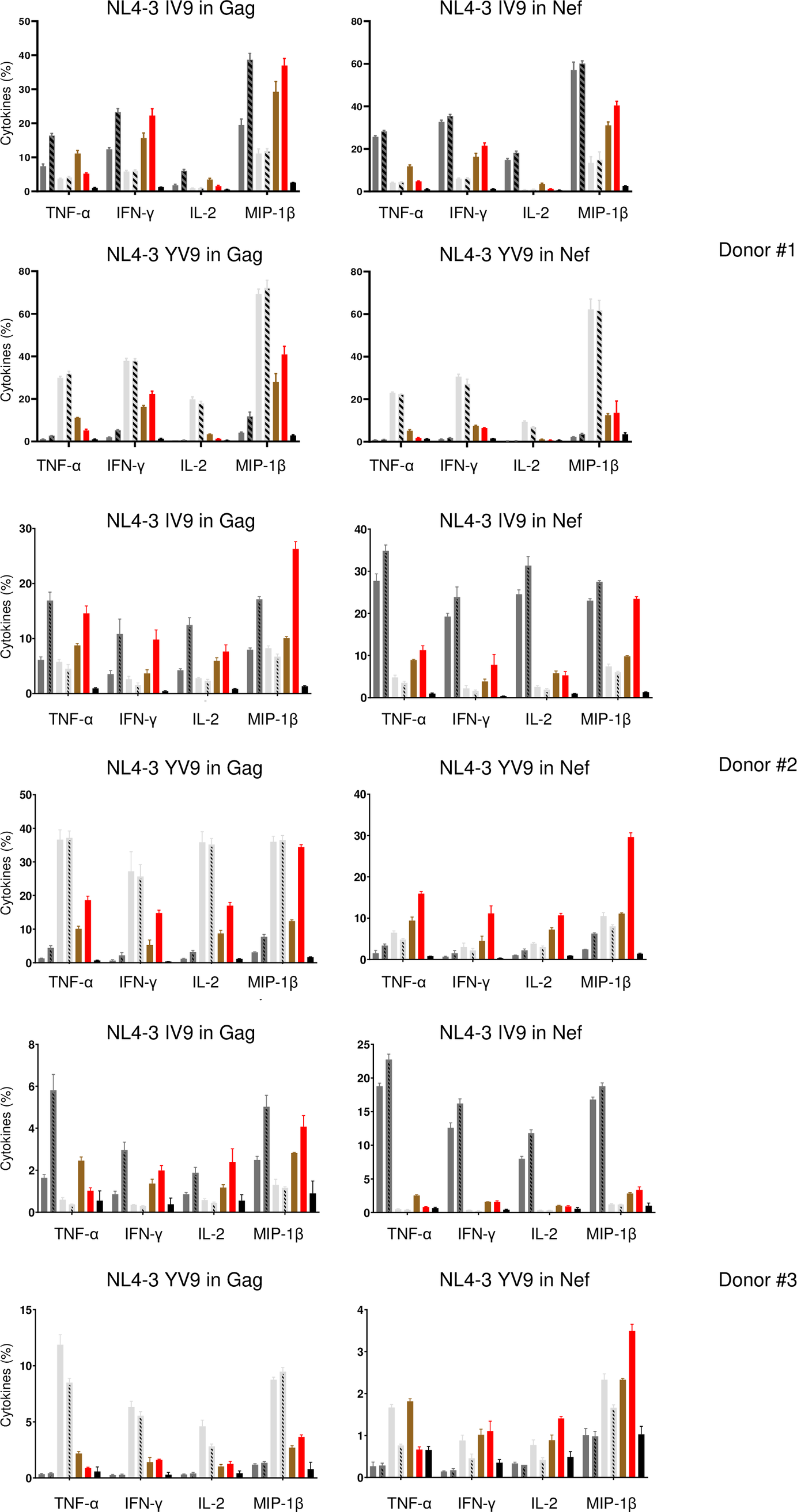

Supplement: S6 Fig — (TIF) [file ppat.1011853.s006.tif]

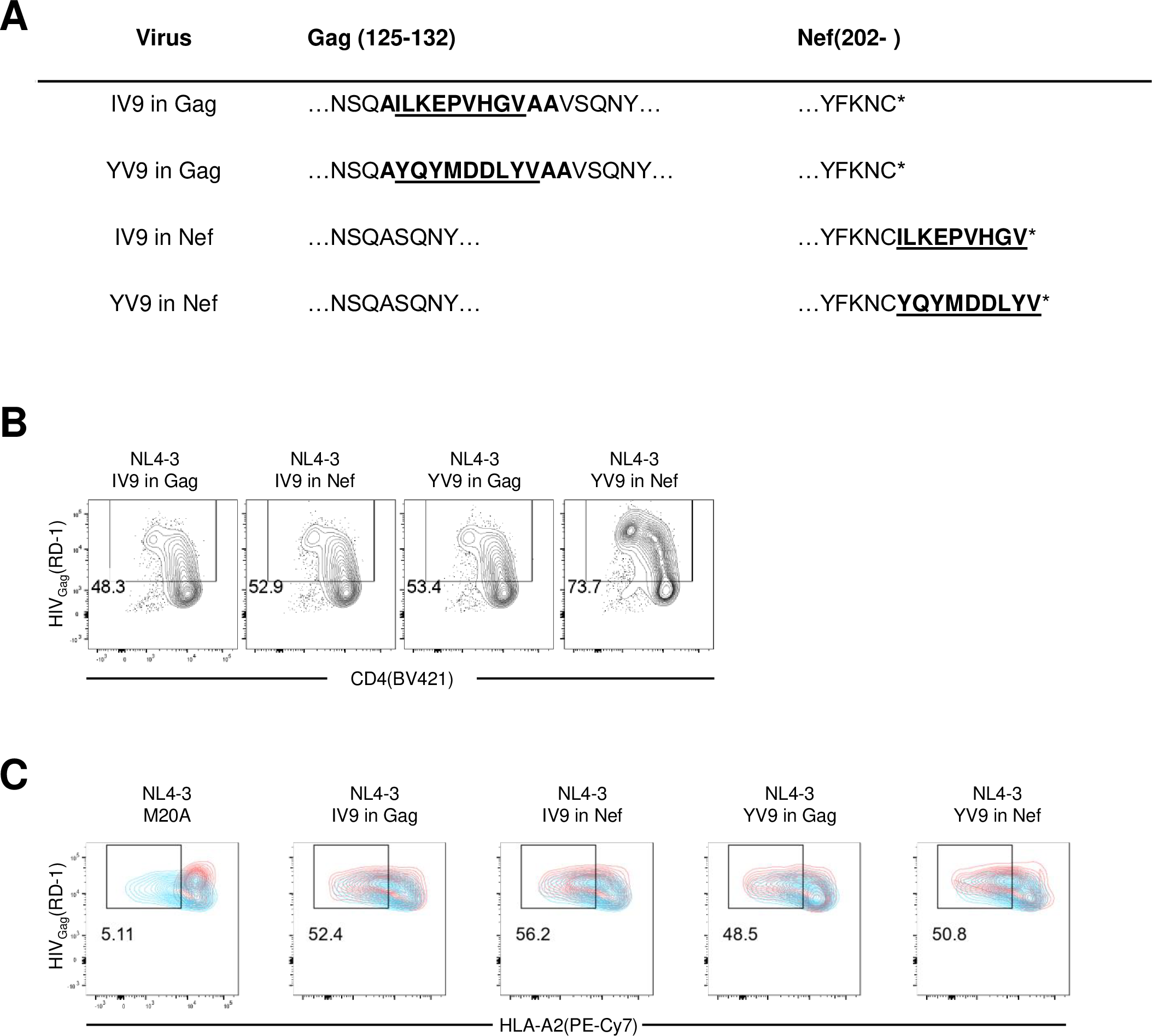

Supplement: S7 Fig — A. Epitope inserted HIVNL4-3 was made by addition of the indicated epitope sequence within pNL4-3 plasmid. Amino acid sequence of the edited spot is shown. B and C. Primary CD4 T cells were activated and infected with HIVNL4-3 WT or indicated mutant viruses on the following day. 4 days after infection, intracellular Gag and surface CD4 expression (B.) or intracellular Gag and HLA-A2 surface staining (C.) was performed. Blue color represents cells infected with HIVNL4-3 WT and red depicts cells infected with the indicated HIVNL4-3 mutant. (TIF) [file ppat.1011853.s007.tif]

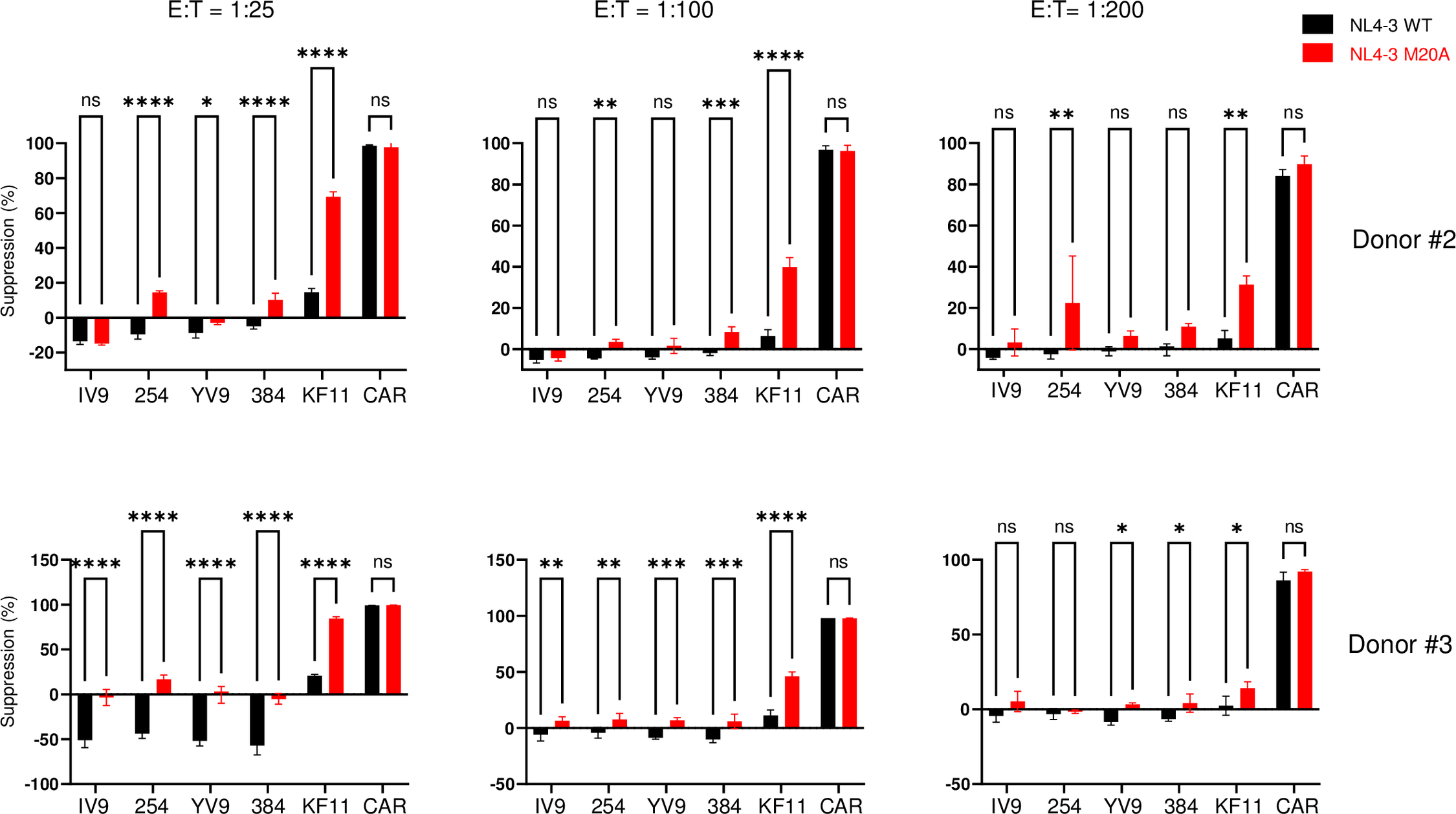

Supplement: S8 Fig — (TIF) [file ppat.1011853.s008.tif]

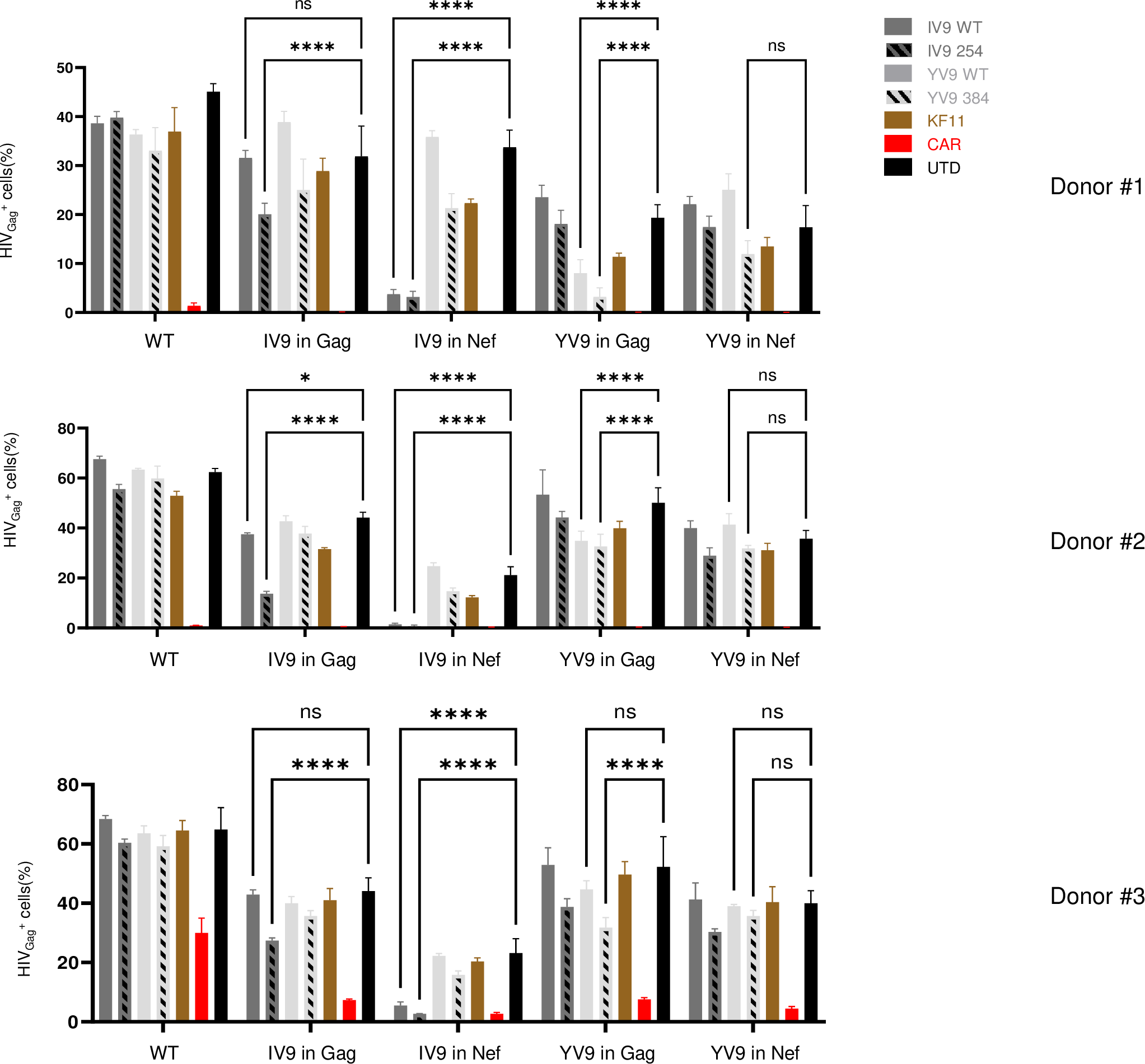

Supplement: S9 Fig — (TIF) [file ppat.1011853.s009.tif]

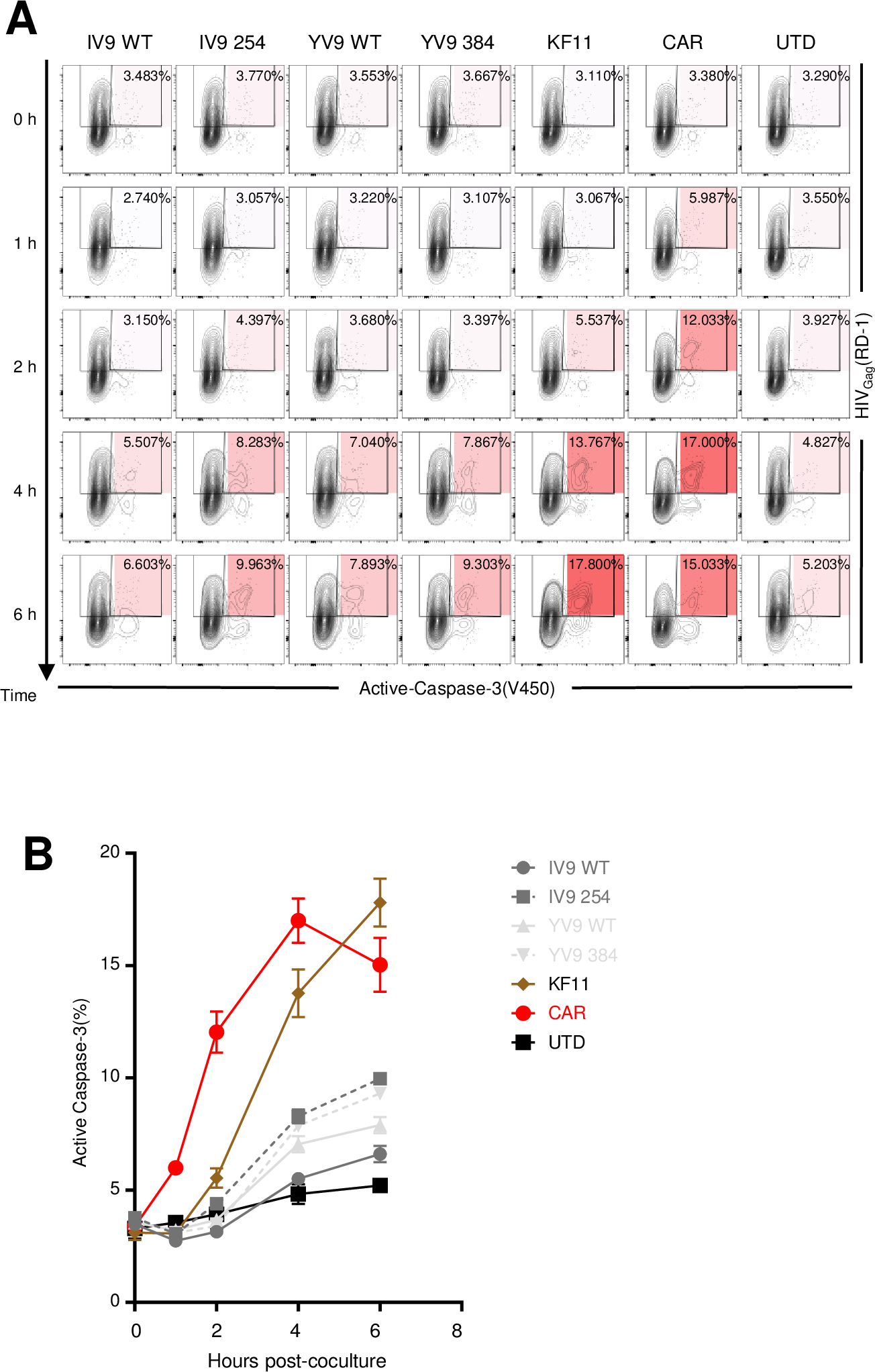

Supplement: S10 Fig — A. Intracellular active Caspase 3 was measured after the coculture of engineered T cells and CD4 T cells infected with HIVNL4-3 Nef M20A for the indicated time (hours). B. Summarized data from A. (TIF) [file ppat.1011853.s010.tif]

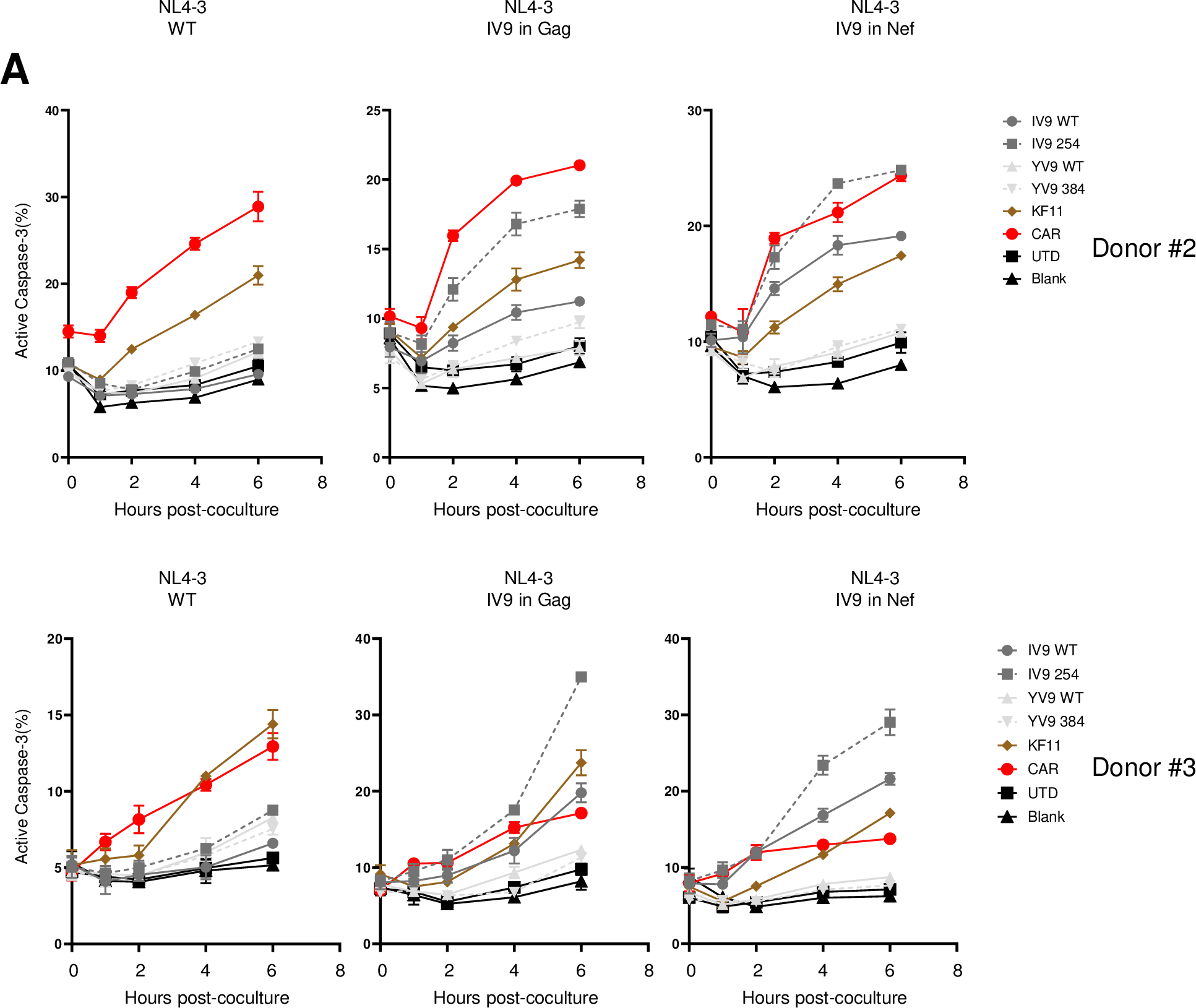

Supplement: S11 Fig — (TIF) [file ppat.1011853.s011.tif]

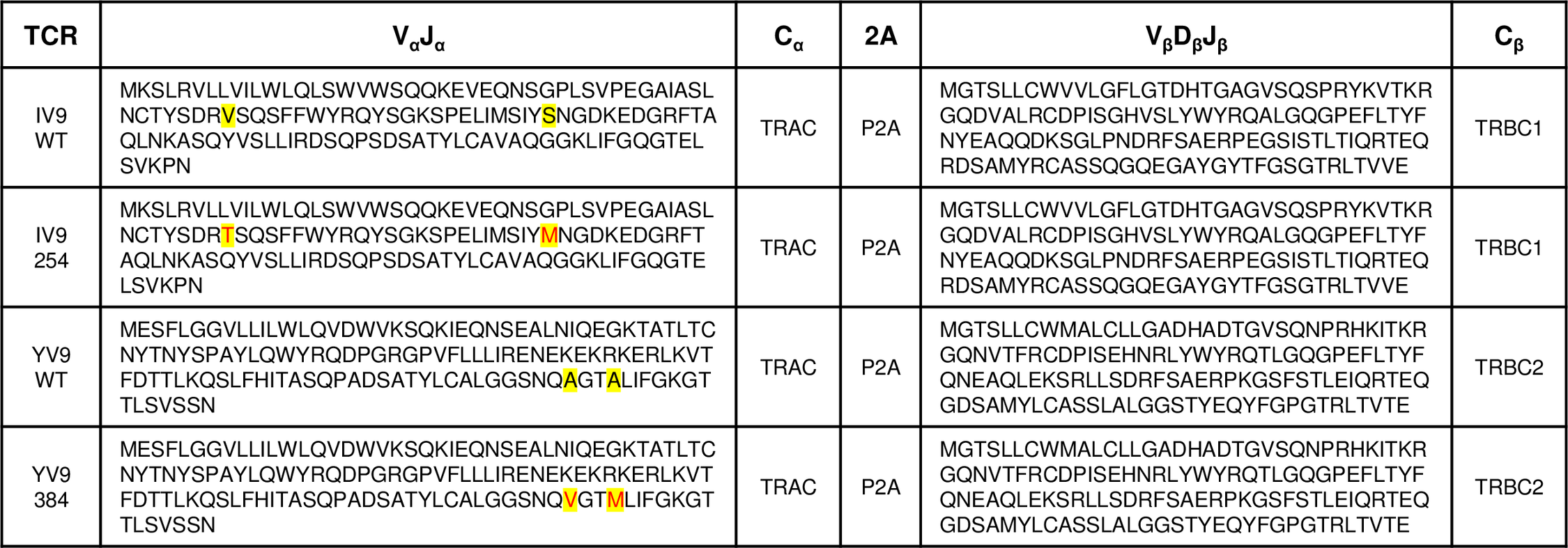

Supplement: S1 Table — (TIF) [file ppat.1011853.s012.tif]
